# Breaking Barriers: Unveiling Sex-Related Differences in Cerebrospinal Fluid Analysis—A Narrative Review

**DOI:** 10.3390/biology13060420

**Published:** 2024-06-05

**Authors:** Raffaella Candeloro, Caterina Ferri, Tiziana Bellini, Maura Pugliatti, Massimiliano Castellazzi

**Affiliations:** 1 Department of Neurosciences and Rehabilitation, University of Ferrara, 44121 Ferrara, Italy; raffaella.candeloro@unife.it (R.C.); tiziana.bellini@unife.it (T.B.); maura.pugliatti@unife.it (M.P.); 2 Department of Neuroscience, “S. Anna” University Hospital, 44124 Ferrara, Italy; caterina.ferri@ospfe.it; 3 University Strategic Center for Studies on Gender Medicine, University of Ferrara, 44121 Ferrara, Italy

**Keywords:** cerebrospinal fluid, blood–cerebrospinal fluid barrier, cerebrospinal fluid analysis, sex-differences

## Abstract

**Simple Summary:**

In recent years, biomedical research has extensively explored gender and sex differences to achieve individualised care. These advancements are yielding new insights across various medical domains. Our review focuses on sex-related disparities in the field of neurological disorders, specifically examining differences observed in certain biomarkers routinely used in cerebrospinal fluid analysis for diagnostic purposes. Our bibliographical research initially indicates that a gender/sex-based approach to data analysis is still relatively uncommon in this field. However, our work highlights a strong consensus among studies thus far that male patients exhibit higher protein content compared to females, likely attributable to differing functionality of the structures responsible for isolating the central nervous system from the bloodstream. Our study aims to prompt the development of sex-specific guidelines for evaluating laboratory parameters of neurological interest, thereby mitigating the risk of overestimation in one sex and/or underestimation in the other. In the era of the search for cost-effective, personalised and precision medicine, these considerations could have a practical impact for the better management of patients with suspected neurological disorders.

**Abstract:**

(1) Background: The recent emphasis on sexual and gender diversity’s impact on human health underscores the need for tailored diagnostic and therapeutic approaches in neurology. The aim of this article is to conduct a narrative review of the available scientific literature on sex differences in cerebrospinal fluid analysis. (2) Methods: The literature search encompassed PubMed databases, focusing on cerebrospinal fluid analysis and sex differences, considering parameters like cerebrospinal fluid protein content, cell count, albumin quotient (QAlb) and intrathecal IgG synthesis. (3) Results: Nine articles from the past two decades were identified, revealing limited research in this area. Males consistently exhibited higher cerebrospinal fluid protein content and albumin quotient values across various pathologies and age groups. Consequently, males more frequently manifested blood–cerebrospinal fluid barrier dysfunction than females. No significant sex differences were observed in cerebrospinal fluid leukocyte count or intrathecal IgG synthesis. (4) Conclusions: This review highlights the dearth of research on sex differences in cerebrospinal fluid analysis, despite consistent findings of higher protein content and albumin quotient values in males. Revisiting current diagnostic thresholds based on sex is crucial for accurate prognosis and personalised treatment strategies in neurological disorders. Moving towards sex-specific approaches in clinical practice is imperative for advancing personalised medicine.

## 1. Introduction

In recent years, there has been a growing emphasis on the exploration of sexual and gender diversity and its impact on human health, an area of study that has frequently been overlooked [1].

In the field of neurology, there are clear differences between males and females, which include various aspects such as the intricate nuances of brain development, the complexities of brain structure, the different functionalities inherent in each gender and the underlying complexities of brain biochemistry [2]. These differences are the basis of clinical implications such as different susceptibilities, different progression, and manifestation across sexes (Table 1) [3,4,5,6,7,8,9,10,11]. These nuanced sex differences highlight the imperative to implement tailored diagnostic methodologies and therapeutic interventions, meticulously tailored to the different neurological landscapes observed between male and female patients, thus ensuring more effective and personalised healthcare outcomes [2]. The first differences to be studied are the anatomical ones, which are the most macroscopic and obvious, especially thanks to imaging [12]. Differences between the two sexes were found in cranial size: men have larger heads than women and a brain volume that is approximately 10% larger [13]. The differential growth of brain volume was very similar to that of the body: men have larger brains throughout life compared to women, due to different early stages of growth [14]. Other studies have shown that a larger skull volume in men means that they have a larger volume of cerebrospinal fluid (CSF) and therefore a larger volume of ventricles and cerebral sulci [15]. Another important difference concerns the different distributions of white matter (WM) and grey matter (GM); a study conducted on 80 patients aged between 18 and 45 years showed that women had more grey matter in the left hemisphere, while men had more WM and CSF [16]. This was already seen in 1997, where it was found that different cranial sizes were dependent on the amount of WM [16]. In the past, it was said that WM was useful matter for information transfer processes, hence the idea that skulls with smaller volumes needed shorter distances for information transfer, and therefore less WM [16]. These anatomical differences underscore the intricate interplay between sex and neurology and highlight the need for tailored diagnostic and therapeutic strategies that recognise and address the different neurological profiles of male and female patients [1,17]. Understanding these macroscopic differences serves as a critical foundation for unravelling the complexities of neurological function and pathology between the sexes, paving the way for more effective and personalised approaches to patient care [1,17].

### 1.1. The Cerebrospinal Fluid Analysis

The CSF is a clear, colourless biological fluid that permeates the entire central nervous system (CNS).

Its composition is like a plasma ultrafiltrate, with important differences in that it has a low protein content and very few cells of the leukocyte lineage (<5 µL) [18]. Both CSF and CNS homeostasis are maintained by the intricate network of barriers within the central nervous system, primarily the blood–brain barrier (BBB) and the blood–cerebrospinal fluid barrier (BCSFB) [19]. These barriers play crucial roles in regulating the exchange of substances between the blood, the brain parenchyma, and the cerebrospinal fluid (CSF), ensuring the optimal functioning and protection of the brain and spinal cord [19]. The BBB is primarily formed of endothelial cells that line the capillaries in the brain [20]. These endothelial cells are interconnected by tight junctions, which create a physical barrier that restricts the movement of substances between the blood and the brain. The BBB mainly separates the blood from the brain parenchyma [20]. The BCSFB separates the blood from the cerebrospinal fluid (CSF), and it consists of specialised epithelial cells in the choroid plexus and the arachnoid membrane. The BCSFB regulates the exchange of substances between the blood and the CSF [21].

The CSF has two important functions: (i) mechanical, whereby it acts as a shock absorber for mechanical stresses associated with variations in head movement, and (ii) biological, whereby it maintains the homeostasis of the entire CNS [22]. The CSF is collected using a mini-invasive procedure called “spinal tap” or lumbar puncture. CSF analysis is of great importance in the study of several diseases that affect both the CNS and PNS, i.e., diseases of a neurodegenerative and neuroinflammatory nature [23].

Physico-chemical analysis of the CSF provides information about its appearance, colour, glucose content, number of circulating cells and protein content [24]. Under normal conditions, no erythrocytes are present in the CSF and only a few cells of the leukocyte lineage and proteins can be counted. An increase in erythrocytes indicates a subarachnoid haemorrhage or a traumatic lumbar puncture, an increase in leukocytes is a sign of inflammation, infection or neoplasia, and an increase in protein is an alarm signal for certain pathological conditions: bacterial meningitis, viral infections, and autoimmune and infectious polyneuropathies [23].

The CSF protein content and the CSF/serum albumin quotient (QAlb) are parameters that are altered in the presence of BCSFB damage/dysfunction [25]. In particular, for decades, the QAlb has been considered the most accurate indicator of BCSFB functionality [26]. Routine CSF analysis also focuses on the detection of intrathecal antibody production, which is generally associated whit inflammatory conditions or infectious diseases. In particular, assessing the presence of CSF-restricted oligoclonal immunoglobulin G (IgG) bands represents the only laboratory method that supports the diagnosis of multiple sclerosis [23,26,27]. To date, CSF examination remains the only tool, together with brain biopsy, that can demonstrate the presence of CNS inflammation [28].

### 1.2. Aim of the Study

Based on the morphological dimorphism and the different distributions of disease in the two sexes, the aim of this article is to conduct a narrative review of the available scientific literature on sex differences in CSF analysis.

## 2. Materials and Methods

The literature search was conducted in December 2023, and several platforms were consulted, including SCOPUS, PUBMED and EMBASE. The keywords “cerebrospinal fluid analysis” were combined with “sex” and “sex differences”. All guidelines related to CSF analysis were also included. English language and the human species were also used as filters. A second round of literature search was conducted just before submitting the manuscript, with the aim of identifying any new articles that could be relevant to this research. During the review, we repeated our search a third time by consulting the previously mentioned databases but found no new results from our search.

The laboratory parameters of the CSF analysis that were considered were (i) the CSF protein content, a non-specific parameter associated with inflammation and an early warning sign [23]; (ii) the CSF cell content (pleocytosis), an indicator of inflammatory/infectious status [23]; (iii) the albumin quotient, a parameter assessing BCSFB function [26]; (iv) intrathecal IgG synthesis, conducted through the “gold standard” of the isoelectric focusing in agarose gel followed by IgG-specific immunofixation, as an indicator of both autoimmune and infectious CNS inflammation [26].

## 3. Results

Our literature search found only nine articles published in the last 20 years (Table 2). This finding highlights how little research has been conducted on this topic. The Results Section 3 of our study includes a comprehensive review of articles focusing on various sex differences, including protein content, cerebrospinal fluid density, barrier permeability, lymphocyte content and IgG synthesis.

### 3.1. Cerebrospinal Fluid Total Protein Content

The CSF total protein content is increased in neurological disorders such as autoimmune diseases, neoplasms, and inflammatory and infectious diseases, and may be associated with changes in white or red blood cell counts [37]. Although the threshold value of 0.45 g/L is the most used worldwide [37,38], regardless of the sex and age of the patients, it has been highlighted that the protein content of the cerebrospinal fluid actually increases with age [25,29]. To reduce this effect of ageing, age-adjusted normal thresholds have been proposed; however, their application has not yet entered routine practice [38].

Sex differences in protein composition have been analysed over time. The first study to do so extracted 20 years of information (1996–2016) from the Ottawa Hospital database [29]. It included 6068 samples, 63% of which were female. This study highlighted the age dependency of CSF-TP, with male patients having higher levels than female patients at all ages (0.38 g/L vs. 0.32 g/L) [29].

Later, in 2020, our team carried out a study on CSF obtained from a population of 1252 patients aged between 18 to 79 years (648 women and 604 men) admitted to the Sant’Anna Hospital in Ferrara in the period between 2010 and 2018 [30]. In addition to confirming the previously observed higher CSF protein content in males compared to females, our study highlighted that this gap remained consistent regardless of the subjects’ age. In fact, when patients were grouped by age, CSF TP was higher in men than in women in all subgroups: males vs. females, (i) 18–30 years (0.34 vs. 0.32 g /L), (ii) 31–40 years (0.44 vs. 0.34 g/L), (iii) 41–50 years (0.45 vs. 0.33 g/L), (iv) 51–60 years (0.51 vs. 0.41 g/L), (v) 61–70 years (0.50 vs. 0.39 g/L), (vi) over 70 years (0.46 vs. 0.40 g/L). The correlation between age, sex and total protein in the cerebrospinal fluid was also analysed: it was seen that age was positively correlated with CSF-TP in both women and men, with a constant gap of 8.5 mg/dL between the two sexes.

Furthermore, that study revealed for the first time that males were more likely than females to receive a laboratory report indicative of high cerebrospinal fluid protein content, regardless of the pathology they were affected by, as a consequence of inherently higher protein levels. This was in fact highlighted by applying both of the most used upper reference limits (URLs): males vs. females, URL = 0.45g/L (50.2% vs. 28.9%) and URL = 0.50 g/L (41.4% vs. 22.1%) [30].

Another recent study performed a comparative analysis of CSF total protein in 419 (77% males vs. 28% females) patients with immune-mediated neuropathies [31].

According to this study, the CSF protein concentration depends on the serum protein concentration, but also on age, CSF flow rate and the type of analysis. Only 62% of the total population had a high CSF total protein concentration.

In another study, the authors’ first analysis showed differences in the correlation with age, but then, the previously performed analyses for male (0.607 g/L) and female (0.477 g/L) patients were re-evaluated. Concordant results for age at diagnosis and lumbar puncture were obtained for patients of both sexes when samples with a high QAlb and normal total protein levels were compared with samples with high total protein levels and a normal QAlb. The same age-dependent effect was seen in samples from female patients when isolated high QAlb values were compared with all samples with high CSF total protein values [31].

The different CSF protein contents in the two sexes could also influence the CSF density itself, a parameter that depends on the protein concentration, glucose content and cell count. The CSF density was assessed in relation to the age, sex, weight and height of the patients. It was observed that only sex had a discernible impact on the values, revealing higher density levels in men (1000.58 g/L) compared to women (1000.49 g/L) [39].

### 3.2. Blood–Cerebrospinal Fluid Barrier Function

According to international guidelines, the CSF-to-serum QAlb ratio can be used to evaluate BCSFB integrity [23,24,26]. Just as was highlighted for CSF protein content, the QAlb also tends to increase with the age of the patients [25].

Recent studies have begun to conduct analyses of sexual dimorphisms in barrier permeability; Parrado-Fernandez and coworkers conducted a study in a sample of 20,000 patients with suspected neurodegenerative or neurological diseases and 335 healthy volunteers aged 1–90 years, in which differences between the two sexes were found in all age groups when patients with albumin levels above 400 mg/dL were excluded. No major sex differences were observed during childhood, puberty or menopause, and age-related changes were more common than sex-related changes [32].

We conducted an analysis on patients admitted to the neurology department; specifically, 1209 people were included (718 women and 491 men—age: 15–88 years), of whom 24.6% had multiple sclerosis, 23.2% had inflammatory neurological diseases (OIND), 24.6% had non-inflammatory neurological diseases (NIND) and 27.6% had no diagnosis [33].

In our population, men had higher QAlb values than women at all ages, and male sex and increasing age were associated with higher QAlb values, regardless of the pathology they were suffering. As a result, men were more likely to have BCSFB dysfunction than women (44% vs. 20.1%), even when stratified by age.

This sex-related difference was also confirmed in a German study on a cohort of 989 patients (545 women and 444 men), all suffering from schizophreniform and affective syndromes [34]. It was found that 18% of the patients had an elevated QAlb compared to the control group; furthermore, a significant difference was found when comparing the two sexes, with a higher number of age-adjusted QAlbs in male patients (26%) compared to females (10%). These QAlb values suggest that the integrity of the blood–brain barrier is more often compromised in men with schizophrenic psychosis. To exclude the coexistence of several neurological diseases in the same patient, a ‘post hoc’ analysis was performed. This was carried out only in the subgroup of individuals without comorbidity. Differences in QAlb emerged, with higher values in male patients than in female individuals [34].

In one study, the QAlb was analysed in 328 patients with amyotrophic lateral sclerosis [3]. Authors found that the QAlb was not associated with age, but with sex, with male patients having higher medain value than female ones. As mentioned above, a study was conducted in January 2024 comparing the total protein-to-albumin ratio in 419 patients with immune-mediated neuropathy [31]. In the total population, 58% had an elevated QAlb. It was also shown that patients with an elevated QAlb and normal total protein were younger at disease onset, at diagnosis and at lumbar puncture than patients with elevated total protein and a normal QAlb. To better understand the different effects of gender and age, a regression analysis was performed for all samples with an elevated QAlb and for samples with elevated CSF total protein, which showed that female patients were less likely to have an elevated QAlb than male patients (median QAlb of females = 7.2; median QAlb of males = 9.5) [31].

### 3.3. Cerebrospinal Fluid Leukocyte Content and Intrathecal IgG Synthesis

Given the distinct barrier functionality between the two sexes and the consequent higher CSF protein content found in males, we further investigated whether these sex-related differences could affect the determination of intrathecal IgG synthesis and the count of leukocytes infiltrating the CNS. Despite male patients exhibiting higher levels of plasma-derived proteins in the CSF, such as albumin and IgG, no differences were observed in the quantitative determination of intrathecal IgG synthesis [35]. Furthermore, no differences between the sexes emerged in the qualitative determination of intrathecal IgG synthesis using the gold standard of searching for CSF-restricted oligoclonal IgG bands, or in the association between CSF leukocyte content and CSF total protein concentration [35,36].

## 4. Discussion

This narrative review firstly highlights how the study of sex differences in laboratory medicine dedicated to neurological patients, or those suspected of having an neurological condition, is still a relatively uncommon approach but has undergone recent evolution. The aspects of CSF analysis examined thus far with a sex-specific approach and the respective differences highlighted are summarised in the Table 3.

Briefly, (i) CSF protein content was higher in males than in females in both neurological patients and hospitalised patients with an undefined diagnosis; (ii) CSF density showed higher values in male subjects who had undergone spinal anaesthesia; (iii) blood–CSF barrier permeability was higher in males than in females in healthy subjects, in patients with SM, Alzheimer’s disease, ALS and immune-mediated neuropathy, and in psychiatric patients; the other way around, no differences were found (iv) in CSF lymphocyte content in hospitalised patients and (v) in intrathecal IgG synthesis in patients with multiple sclerosis and clinically isolated syndrome.

In general, there is complete agreement among the few published works that male subjects have a higher CSF protein content and QAlb values compered to females. Both parameters are increased in the presence of an impaired BCSFB [23,26,40]. The fact that this condition is more prevalent in males, regardless of the pathology from which the patients are suffering, highlights a first important doubt about the applicability of the current thresholds of normality of both these parameters.

Taken together, all works included in this narrative review suggest the need to review the thresholds used for the assessment of QAlb and CSF total protein according to the patient’s sex to avoid potential underestimation of blood–CSF barrier function in women and/or its overestimation in men. It is important to note that in 2017, McCudden and colleagues proposed different thresholds for CSF protein content between males and females [29]. We applied these thresholds to our neurological population and showed a reduction, but not complete elimination, of the sex gaps [30]. However, to date, no other studies have replicated this approach.

None of the studies included in this review had mechanistic ambitions, so it is not possible for us to draw conclusions about the causes of this sex gap. One hypothesis as to the cause of these sex-related differences is anthropometric dimorphism between males and females, resulting in differences in height, weight and body mass index. Moreover, an effect of the “rostro-caudal gradient”, i.e., the distance between the ventricles and the lumbar subarachnoid space, where the protein content seems to increase, has been hypothesised [41]. The hypothesis that sex differences in body height, resulting in a greater spine length and consequently an increased distance for cerebrospinal fluid (CSF) flow in males compared to females, has been posited as a potential explanation for the observed disparity in QAlb values between the sexes [34]. However, our recent study revealed that even after controlling for patients’ height, the independent association of QAlb and CSF TP values with sex remains statistically significant [42].

Moreover, possible effects of body weight and body mass index have also been suggested [43]. In heavier patients, the accumulation of epidural fat may contribute to a decreased CSF flow rate, leading to an elevation in both the QAlb and total protein concentrations [43]. In our study, upon examining both males and females collectively, we noted a positive correlation between weight and BMI with QAlb and, to some extent, with protein concentrations in the CSF [44]. However, this correlation dissipated in sex-stratified analyses and multivariable regressions. It is noteworthy that while variables such as age and sex exhibited independent associations with QAlb and total cerebrospinal fluid protein, weight and BMI did not. Consequently, it appears that the disparities in CSF QAlb and TP concentrations between sexes cannot be solely attributed to variances in height, weight or BMI, as the association between sex and these CSF variables persisted even after adjusting for anthropometric factors [44]. Moreover, the absence of associations between QAlb and CSF protein concentrations with BMI was further confirmed in a multi-comparison analysis in males and females grouped by BMI weight status. No differences emerged when comparing underweight, normal weight, overweight and obese subjects in the two sexes [42].

Considering this lack of evidence on the role of anthropometric differences between the two sexes, we cannot exclude a role of hormonal factors in the parameters covered by this review. Sex-specific disparities in QAlb values and CSF total protein concentrations may stem from various mechanisms. For instance, the female hormone 17β-estradiol [45] could influence the expression of enzymes implicated in BCSFB breakdown [46,47,48], potentially exerting a protective effect on the BCSFB [49]. Intriguingly, since sex-specific QAlb values remain consistent throughout puberty and menopause, it suggests that both genetic predisposition linked to sex chromosomes and hormonal factors play crucial roles in this phenomenon [32,50].

Taking into account all the results obtained from the studies, the differences in the following biomarkers between the two sexes were calculated and are shown in Table 3: (i) protein content; (ii) barrier permeability, except for the study by Parrado, in which the differences in QAlb for the two sexes are not reported; (iii) lymphocyte content; and (iv) intrathecal IgG synthesis. No differences were found for the latter two biomarkers. Despite the general agreement on the main results, the sex-related differences showed heterogeneity upon comparing the studies each other, and this could mainly be due to the different populations analysed.

The data collected in our review showed that biomarkers such as CSF protein and QAlb have different distributions between the two sexes. It is interesting to note that these differences are found in very different diseases, both pathogenetically and epidemiologically, which may indicate that these differences may be physiological and not disease-related. In fact, the differing distribution of biological data between the sexes (Table 3) does not align with the clinical and epidemiological differences observed between men and women (Table 1). 

It would be interesting in the future to conduct further research for other biomarkers as well, such as Tau proteins and amyloid beta cytokines and chemokines, to give a comprehensive view of the topic. A non-negligible aspect is the geographical origin of the data published so far, coming mainly from Europe and Canada. Therefore, despite the agreement between the results produced, the possible effects of ethnicity and geographical distribution should be investigated in future studies.

The main limitation of this review is the small number of articles in the literature, which suggests how unexplored this field still is. Nevertheless, it can be seen that not all of them had mechanistic aims and that they all agreed that both protein content and QAlb were higher in men than in women. Another limitation is the lack of healthy populations in all the included studies, except for the study by Parrado and colleagues [32]. This is mainly due to the invasive nature of a lumbar puncture, which is mainly performed for diagnostic or therapeutic purposes. We would also like to underline how little this topic is addressed in the literature, as can be seen from the number of studies found, compared to the impact it has on routine clinical practice.

## 5. Conclusions

The present review brings together, for the first time, all available evidence regarding sex-related differences in the biochemical parameters of CSF analysis. Despite the absence of the widespread adoption of a sex-specific approach in clinical practice, the existing literature unanimously underscores that irrespective of age or underlying pathology, males exhibit higher QAlb values and CSF protein concentrations compared to females. Consequently, males more frequently manifest BCSFB dysfunction than females. This evidence underscores the necessity of reassessing the current QAlb and CSF protein concentration thresholds based on the sex of individuals undergoing lumbar puncture, and we would also like to point out how little this topic is addressed in the literature, as can be seen from the number of studies found, which prompts us to point out, given the results reported, how important it is. Failing to do so risks overestimating these parameters in one sex while underestimating them in the other. In an era of aspiration to achieve cost-effective, personalised or precision medicine, such considerations have practical significance for sex-specific prognosis in neurological disorders.

## Figures and Tables

**Table 1:** Sex-related differences in neurological diseases.

	Men	Women	References
Amyotrophic lateral sclerosis	High prevalence	Faster progression	[3]
Alzheimer’s	Lower prevalence	High prevalence and faster clinical decline	[4]
Parkinson’s	High prevalence	Slower progression	[5]
Multiple sclerosis	Lower prevalence	High prevalence	[6,7]
Depression and anxiety	Lower prevalence	High prevalence	[8,9]
Attention-deficit hyperactivity	High prevalence	Lower prevalence	[10]
Autism spectrum disorders	High prevalence	“Camouflage” effect (masking symptoms)	[11]

**Table 2:** Biomarker-specific articles.

Biomarker	References	Title
Protein content	McCudden, C.R. et al. [29]	Cerebrospinal Fluid Total Protein Reference Intervals Derived from 20 Years of Patient Data.
	Castellazzi M. et al. [30]	Sexual dimorphism in the cerebrospinal fluid total protein content.
	Seeliger, T. et al. [31]	Comparative analysis of albumin quotient and total CSF protein in immune-mediated neuropathies: a multicenter study on diagnostic implications.
Albumin quotient	Parrado-Fernandez, C. et al. [32]	Evidence for sex difference in the CSF/plasma albumin ratio in ~20,000 patients and 335 healthy volunteers.
	Castellazzi, M. et al. [33]	Increased age and male sex are independently associated with higher frequency of blood–cerebrospinal fluid barrier dysfunction using the albumin quotient.
	Meixensberger, S. et al. [34]	Sex difference in cerebrospinal fluid/blood albumin quotients in patients with schizophreniform and affective psychosis.
	Seeliger, T. et al. [31]	Comparative analysis of albumin quotient and total CSF protein in immune-mediated neuropathies: a multicenter study on diagnostic implications.
	Verde, F. et al. [3]	Relationship between cerebrospinal fluid/serum albumin quotient and phenotype in amyotrophic lateral sclerosis: a retrospective study on 328 patients
Lymphocyte content	Castellazzi, M. et al. [35]	Sex-Related Differences in Cerebrospinal Fluid Plasma-Derived Proteins of Neurological Patients.
IgG intrathecal synthesis	Castellazzi, M. et al. [36]	The Sexual Dimorphism in Cerebrospinal Fluid Protein Content Does Not Affect Intrathecal IgG Synthesis in Multiple Sclerosis.

**Table 3:** Sex differences in cerebrospinal fluid (CSF).

Biomarker	Differences Male vs. Female	*p* Value	Population	References
Protein content	0.038 mg/L vs. 0.032 mg/L	<0.00001	Neurological patients with nature to be determined	[29]
	460 vs. 370 mg/L	<0.0001	Hospitalised patients with nature to be determined	[30]
	607 vs. 477 mg/L	<0.0001	Patients with immune-mediated neuropathy	[31]
CSF density	1.00058 g mL−1 vs. 1.00049 g mL−1	<0.024	Patients receiving spinal anaesthesia	[39]
Albumin quotient	NA	0.01	Neurological patients and healthy patients	[32]
	5.22 vs. 4.10	<0.0001	Multiple sclerosis	[33]
	9.22 vs. 6.68	0.0172	Inflammatory neurological diseases	
	6.34 vs. 5.09	<0.0001	Non-inflammatory neurological diseases	
	8.48 vs. 4.89	<0.0001	Neurological patients with nature to be determined	
	6.06 vs. 5.29	<0.001	Psychiatric patients	[34]
	6.92 vs. 5.19	<0.001	Alzheimer’s	[34]
	6.8 vs. 5.1	<0.0001	Patients with amyotrophic lateral sclerosis	[3]
	9.5 vs. 7.2	<0.0001	Patients with immune-mediated neuropathy	[31]
IgG intrathecal synthesis	53.2 vs. 60.6% (IgG index)	0.2387	Multiple sclerosis patients	[36]
	48.6 vs. 56.7% (intrathecal fraction)	0.1304		
	86.2 vs. 86.7% (CSF IgG OCBs)	0.4351		
	40.6 vs. 45.6% (IgG index)	0.6642	Clinically isolated syndrome	
	34.4 vs. 43.9% (intrathecal fraction)	0.5002		
	93.8 vs. 89.5% (CSF IgG OCBs)	0.7059		

NA: not available; OCBs: oligoclonal bands.

## Data Availability

Not applicable.
